# Physicians' Knowledge and Practices Regarding Asthma in Jordan: A Cross-Sectional Study

**DOI:** 10.3389/fpubh.2021.712255

**Published:** 2021-08-31

**Authors:** Eman Z. Dahmash

**Affiliations:** Department of Pharmaceutical Sciences and Clinical Pharmacy, Faculty of Pharmacy, Isra University, Amman, Jordan

**Keywords:** practice, physicians, knowledge, Jordan, healthcare professionals, asthma

## Abstract

**Objectives:** Asthma is a chronic non-communicable disease that causes significant morbidity and mortality and requires ongoing clinical care. Appropriate knowledge by physicians is vital in the management of asthma. Therefore, this study aims to explore and identify the gaps in physicians' knowledge and practices concerning the management of asthma.

**Methods:** A cross-sectional study using an online survey was conducted in Jordan to understand the gap in knowledge and practices in the management of asthma among physicians. A previously validated questionnaire was employed, the Physicians' Practice Assessment Questionnaire (PPAQ). The knowledge questions based on the Global Initiative for Asthma (GINA) guidelines were used to assess knowledge and practice among the study's participants. Predictors of good practice were identified using logistic regression.

**Results:** A total of 271 physicians participated in this survey. The overall knowledge among physicians scored above 78%. However, gaps were pertinent to identifying the signs of asthma attacks that accounted for 61.9% of the participants, whereas only 67.6% of the physicians knew the drugs used for the management of asthma. The study revealed alarming results when practices were assessed, with the overall percentage of physicians applying the required practices did not exceed 57.1 ± 25.7%. Logistic regression analysis to determine predictors of good practice showed that out of several independent variables, physicians who see 6–10 asthma patients per day are five times more likely to follow the guidelines' recommendations in their practice; senior physicians (>50 years old) and those who see 1–5 asthma patients daily are around two times more likely to follow the guidelines (*p* 0.001).

**Conclusions:** The findings of this study identified the need to transform knowledge into practice. This could be achieved through professional education and constant reminders to physicians in a simple form, as well as a clinical audit of practice. There is a need for novel knowledge transfer approaches to induce behavioral and practice change toward the management of asthma.

## Introduction

Asthma is one of the main non-communicable diseases affecting more than 339 million people worldwide and is most commonly prevalent among children (1). Although asthma is more prevalent in developed countries, most deaths that are related to asthma are attributed to low and lower-middle-income countries. The World Health Organization (WHO) reports (2016) revealed that the annual asthma-related global deaths exceeded 400,000. To provide a better representation of the burden of the disease, the disability-adjusted life year (DALY) for asthma is used. In 2016, the DALY due to asthma exceeded 24.8 million (2, 3). The global burden of asthma is continuing to rise with the increase in asthma symptoms' prevalence (4, 5). In 2016, 1.8 million people visited emergency departments for asthma-related care, and within the same year, 189,000 people were hospitalized because of asthma (6).

In Jordan, the prevalence of physician-diagnosed asthma ranged between 8.8 and 9.5% (7). A study by Abu-Ekteish and colleagues revealed that the prevalence of asthma in Jordan has doubled in the last 10 years (7). In 2015, the prevalence of asthma in Jordan among the elderly reached an alarming figure of 10% (8, 9). Moreover, in 2018, a study showed that asthma was diagnosed in 6.2% of adolescents in northern Jordan (10). Yet, studies and official reports indicate that asthma is poorly managed. The WHO reports disclosed that asthma is under-diagnosed and under-treated. “*It creates a substantial burden to individuals and families and often restricts individuals' activities for a lifetime*.” (2). Poor control of asthma could be attributed to several barriers including those during diagnosis, treatment, and follow-up (11). A lack of standard diagnostic criteria and equipment are the key challenges encountered in the diagnosis stage. Whereas, insufficient access to medications and healthcare services, poor communication, and the high cost of medications are some of the critical challenges in the treatment and follow-up phases. During the follow-up, the lack of patient education led to improper use of devices and is one of the main contributors to poor control of asthma (2, 11).

Although there is no cure for asthma, medications and proper management can enable patients with asthma to enjoy a good quality of life. Recurrent asthmatic attacks can result in sleeplessness, fatigue, low levels of activity, and absenteeism from work or school (2). Despite the advances in the understanding of asthma's pathogenesis and treatment, it remains a significant public health concern with considerable morbidity and mortality worldwide (7). Physicians' knowledge and practice have been recognized as key elements that affect disease outcomes. However, there is little information on specific practice differences related to asthma management (12). A recent study by Price et al. (13) reported that poor control of asthma is attributed to gaps between evidence-based recommendations and practice (13).

In general, around two-thirds of patients suffering from asthma are receiving care from a primary healthcare setting. Studies have reported that medication use, allergen control, and continuous education can be key strategies to reduce the morbidity and healthcare costs associated with asthma (12, 14). However, it is important to determine the knowledge and practices of physicians in the management of asthma as the main contributor to the good clinical outcome of the disease (15, 16). Therefore, this study aims to explore the knowledge and practice among physicians in the management of asthma in Jordan, and to identify the key gaps.

## Methods

### Study Design and Study Population

A cross-sectional study by means of an online survey was conducted in Jordan between 15^th^ October and 30^th^ November 2020 to explore the knowledge and practice of physicians concerning asthma management in Jordan. Participants were given 2 weeks to respond. A reminder was send and additional 2 weeks were allowed for responses.

### Sampling Strategy

A convenient sample of eligible participants was invited to participate in the study. Physicians were invited to participate through social media platforms (Facebook, WhatsApp, and emails). All participants voluntarily participated in the study and were thus considered exempt from written informed consent. The study's aim and objectives were clearly explained at the beginning of the survey questionnaire. The inclusion criteria were (a) physicians living currently in Jordan, and (b) physicians practicing medicine in Jordan. Further, physicians not seeing any asthmatic patients in their clinics were excluded from the practice section.

### The Assessment Questionnaire

The assessment questionnaire was made of three sections. The first section focused on participants' background and demographic information (age, gender, work experience, specialty, and job description). Additionally, all participants were asked about the average number of patients seen at their clinic per day and the average number of asthma cases seen at the clinic per day. The second section addressed knowledge, which was based on studying the relationship between knowledge and the quality of asthma management by Adeniyi et al. with permission (17). This section comprised of 10 knowledge questions, each question was based on a yes or no answer. Three questions in the knowledge section under “nature of the disease” (items numbered 1, 3, and 4) were negatively worded and thus, were reversely scored during the analysis, where “NO” meant “true answer” and “YES” meant “wrong answer.” The third section was based on the Physicians' Practice Assessment Questionnaire (PPAQ), which was designed for use with physicians to conduct a self-assessment of the implementation of asthma management guidelines (18). The asthma PPAQ entailed 14 key actions that are in line with the international guidelines (18, 19). The questions addressed practice in relation to diagnosis, assessment, treatment, and follow-up. The tool asked physicians about the degree to which each action is applied to his/her practice. The participants' responses ranged from 0 to 100%, and the average number of doctors who were confirmed as completing each section was calculated. The cut-off score for good practice was set at 57% (which is the average practice score value for the whole study sample). Overall, the use of a pre-existing scale has the advantage of using a validated and tested instrument, which increases the reliability of its measure.

### Ethical Approval

All study participants gave their informed consent for inclusion before they participated in the study. The study protocol was approved by the Research Ethics Committee of the Faculty of Pharmacy at Isra University (PH – 2021 – 14).

### Statistical Analysis

Data was analyzed employing SPSS software, version 25. Descriptive statistics were used to describe the participants' demographic characteristics. Data were reported as mean ± standard deviation (SD) for normally distributed variables, while categorical data were reported as percentages (frequencies). Logistic regression analysis (univariate analysis) was used to determine predictors associated with good practice. Good practice was defined as the total percentage of asthma practices of 57% and above (which is the mean value). A confidence interval of 95% (*p* < 0.05) was applied to represent the statistical significance of the results and the level of significance was assigned as 5%. The questionnaire was designed to prevent the submission of incomplete forms, hence there was no missing data.

## Results

### Background and Demographics

A total of 271 physicians participated in the study. Table 1 details the baseline characteristics of the participants. The majority of participants (*n* = 169, 62.4%) were males, the largest proportion of the participants were aged between 30 and 34 years (*n* = 98, 36.2%), and with work experience between 6 and 10 years (*n* = 100, 36.9%). The majority of physicians hold specialties that are entailed in managing asthma in Jordan (*n* = 159, 58.7%); whereas more than half (*n* = 143, 52.8%) are either specialists or consultants. Around one-third of the participants (*n* = 87, 32.1%) examine an average of 11–20 patients per day, followed by more than 30 patients which account for 24.4% of participants. When physicians were asked about the average number of asthma cases seen at the clinic daily the majority reported that it is between 1 and 5 patients per day (*n* = 171 63.1%).

**Table 1:** Demographic and practice' characteristics for the study participants.

**Variable**	**Frequency (%)**
**Age (categories)**	
25–29 years	41 (15.1)
30–34 years	98 (36.2)
35–39 years	45 (16.6)
40–44 years	26 (9.6)
45–49 years	10 (3.7)
50 years and above	51 (18.8)
**Gender**	
Male	169 (62.4)
**Work experience (years)**	
<5 years	64 (23.6)
6–10 years	100 (36.9)
11–15 years	39 (14.4)
16–20 years	17 (6.3)
More than 20 years	51 (18.8)
**The average number of patients seen at the clinic per day**	
1–10 patients	58 (21.4)
11–20 patients	87 (32.1)
21–30 patients	60 (22.1)
More than 30 patients	66 (24.4)
**The average number of asthma cases seen at the clinic per day**	
Zero	58 (21.4)
1–5 patients	171 (63.1)
6–10 patients	32 (11.8)
11–20 patients	6 (2.2)
More than 20 patients	4 (1.5)
**Specialty**	
Pulmonologist	10 (3.7)
Internal medicine specialist	22 (8.1)
Family medicine specialist	32 (11.8)
Pediatrician	33 (12.2)
Emergency physician	9 (3.3)
General practitioner	53 (19.6)
Other specialty	112 (41.3)
**Job description**	
General practitioner	58 (21.4)
Resident	70 (25.8)
Specialist	89 (32.8)
Consultant	54 (19.9)

### Knowledge About Asthma

Table 2 presents the physicians' knowledge about asthma from the questionnaire. The results revealed variations according to the domain. Overall, 78.3% of physicians demonstrate good knowledge about asthma. With the asthma knowledge questions, the three domains: *nature of the disease, symptoms of asthma, and trigger of asthma* had averages of correct responses that exceeded 80% (89.5, 89.3, and 84.6%, respectively). The least number of physicians recording a correct response was for question number “8,” “*sign of asthma attack*,” accounting for 61.9%. Furthermore, only 67.6% of physicians knew the drugs used for the management of asthma.

**Table 2:** Frequency of the study participants answering the asthma knowledge questions right.

**No**.	**Knowledge item**	**Frequency (Yes %)**
	***Nature of the disease***	
**1**	Asthma is not a chronic inflammatory disorder of the airways	47 (17.3) ǂ
**2**	Symptoms of asthma occur or worsen at night, awakening the patient	248 (91.5)
**3**	Symptoms of asthma does not have a seasonal pattern	33 (12.2) ǂ
**4**	Family history is not relevant	17 (6.3) ǂ
**5**	Asthmatic chronically inflamed airways are usually hyper responsive	249 (91.9)
**6**	***Which of the following is/are symptoms of asthma?** (more than one answer is possible)*	
	Cough (worse particularly at night)	246 (90.8)
	Recurrent wheeze	262 (96.7)
	Recurrent difficulty with breathing	246 (90.8)
	Recurrent chest tightness is possible	214 (79.0)
**7**	***Which of the following is a trigger of asthma?** (more than one answer is possible)*	
	Animal fur	245 (90.4)
	Aerosol	209 (77.1)
	Changes in temperature	206 (76.0)
	Domestic dust	254 (93.7)
	Drugs	224 (82.7)
	Exercise chemicals	205 (75.6)
	Pollen	251 (92.6)
	Respiratory (viral) infections	243 (89.7)
	Smoke	265 (97.8)
	Strong emotional expression	191 (70.5)
**8**	***Which of the following is used in diagnosing asthma?** (more than one answer is possible)*	
	Spirometer	246 (90.8)
	Peak flow meter	216 (79.7)
	Chest radiography	123 (45.4)
**9**	***Which of the following a sign of asthma attack:** (more than one answer is possible)*	
	Cyanosis	189 (69.7)
	Fast pulse rate	190 (70.1)
	Duration of attack	124 (45.8)
**10**	***Which of the following is/are drugs for the management of asthma:** (more than one answer is possible)*	
	Oral prednisolone	235 (86.7)
	Salbutamol	264 (97.4)
	Adrenaline	130 (48.0)
	Cromolyn	147 (54.2)
	Antibiotics	82 (30.3)
	Intravenous hydrocortisone	205 (75.6)
	Intravenous aminophylline	174 (64.2)
	Ipratropium bromide	199 (73.4)
	Intranasal oxygen	212 (78.2)

*ǂ The correct answer to this question is “NO” hence in the calculations of the overall percentage the result will be subtracted from 100*.

### Practice in Asthma Management

The physicians' practice results are presented in Table 3. The average percentage of physicians who manage asthma patients according to the guidelines did not exceed 57% (SD: 25.7%). The majority of physicians (66.1%) provide smoking cessation counseling and/or recommend cessation measures despite a high standard deviation of 36.2%. Only 44% of physicians provide a written referral for asthma education. Almost two-thirds of physicians check for treatment adherence at each visit, refer their patients to a specialist if the asthma diagnosis is uncertain, and schedule regular follow-up appointments. Physicians' compliance with guidelines was low (45.3%). With respect to confirming the diagnosis by pulmonary function tests, only 52% reported that they do so using either spirometry and bronchodilator reversibility or broncho-provocation.

**Table 3:** Asthma practices' characteristics for the study participants.

**No**.	**Practice's item**	**Average percentage of physicians who confirmed applying it**
**1**	Confirm diagnosis by pulmonary function tests (either spirometry and bronchodilator reversibility or broncho-provocation	52.0% (± 35.6%)
**2**	Provide written referral for asthma education	44.4% (± 33.3%)
**3**	Provide a written action plan for exacerbation management	48.8% (± 34.3%)
**4**	Assess inhaler technique (or refer to asthma educator) at each visit	55.3% (± 36.1%)
**5**	Identify environmental triggers/inducers	61.2% (± 34.6%)
**6**	Provide smoking cessation counseling and/or recommend cessation measures	66.1% (± 36.2%)
**7**	Prescribe an inhaled corticosteroid (ICS) as initial maintenance therapy	58.1% (± 37.7%)
**8**	Prescribe an inhaled ICS and a long-acting beta2-agonist (LABA) when asthma is not controlled by ICS low dose alone	60.8% (± 36.7%)
**9**	Check for treatment adherence at each visit	64.1% (± 34.1%)
**10**	Use the Canadian Thoracic Society (CTS) control criteria or the Global Initiative for Asthma (GINA) guidelines to assess patient's asthma control	45.3% (± 35.7%)
**11**	Address patients' concerns about disease/treatment	63.8% (± 35.3%)
**12**	Refer to a specialist because asthma is difficult to control	50.8% (± 35.7%)
**13**	Refer to a specialist if the asthma diagnosis is uncertain	64.3% (± 35.9%)
**14**	Schedule regular follow-up appointments	64.5% (± 35.9%)
	Average	57.1% (± 25.7%)

### Predictors of Better Asthma Practice

Logistic regression analyses to predict independent variables' contribution to better asthma practice among physicians are listed in Table 4. Good practice was defined as the total percentage of asthma practice (Table 3) of 57% and above (mean value). The results showed that the physicians' age was a significant predictor that affected their practice. Physicians within the age group of 35–39 years had the worst practice results, with 53% more likely to not carry out all the recommended asthma practices; while seniors are two times more likely to apply the guidelines' recommendations through their practice. Furthermore, physicians who see 6–10 asthma patients per day are five times more likely to follow the guidelines' recommendations in their practice, and those who see 1–5 asthma patients daily are around twice more likely to follow the guidelines, as shown in Table 4.

**Table 4:** Logistic regression analysis (univariate analysis) to identify factors affecting asthma practices.

**Variable**	**Odds ratio (95%CI)**
**Age (categories)**	
25–29 years (Reference)	1.00
30–34 years	0.79 (0.48–1.30)
35–39 years	0.47 (0.24–0.90)*
40–44 years	1.90 (0.79–4.52)
45–49 years	0.79 (0.22–2.79)
50 years and above	1.96 (1.03–3.75)*
**Gender**	
Male (Reference)	1.00
Female	1.31 (0.79–2.15)
**Work experience (years)**	
<5 years (Reference)	1.00
6–10 years	0.79 (0.48–1.29)
11–15 years	0.57 (0.29–1.12)
16–20 years	2.73 (0.87–8.61)
More than 20 years	1.96 (1.02–3.74)*
**The average number of patients seen at clinic per day**	
1–10 patients (Reference)	1.00
11–20 patients	1.47 (0.87–2.47)
21–30 patients	1.05 (0.59–1.87)
More than 30 patients	1.30 (0.74–2.29)
**The average number of asthma cases seen at clinic per day**	
1–5 patients (Reference)	1.00
6–10 patients	5.01 (1.87–13.44)**
11–20 patients	0.79 (0.16–4.00)
More than 20 patients	2.41 (0.25–23.49)

Significant predictor (*p < 0.05, **p < 0.01).

## Discussion

This study was aimed at identifying potential gaps in knowledge and practice from physicians in Jordan regarding asthma. The findings of this work revealed that the overall level of asthma knowledge is acceptable, with the overall percentage of physicians that demonstrate good knowledge just above 78%. The main gaps in knowledge were pertinent to the use of chest radiography for the diagnosis of asthma, with only 45.5% of physicians reported the need to do the test to exclude other diseases. Similar findings were reported by (17) where only 61% of physicians employed chest radiographs upon diagnosing asthma (17). Although national and international guidelines support the use of chest imaging to exclude comorbidities or the assessment of complications (20), this seems to be not routinely done. While knowledge rates were encouraging when it comes to the nature of the disease and triggers of asthma, <70% of physicians knew the signs of asthma. Therefore, the next gap of knowledge is pertinent to the signs of an asthma attack with the results' percentages below 75%. Overall, the physicians' knowledge was low in this domain and this is in line with several studies that recommend patients be educated about the signs of attack and what needs to be done in that situation (17). Only 30.3%, of the respondents confirm the use antibiotics; while 48% agree on the use of adrenaline in the management of asthma, despite the recommendations of the GINA (21, 22). This lack of knowledge of specific guidelines-related therapy recommendations will adversely affect the clinical outcome of patients, particularly in the management of exacerbations.

Asthma is associated with a substantial social and economic burden (23) due to direct (e.g., hospitalization, and medications) and/or indirect costs (e.g., sickness/absence days and early death). In 2013, the costs for treating a patient with controlled asthma were estimated at about EUR 509; while for patients with uncontrolled asthma the cost can reach up to EUR 2,281 per patient (14). For this reason, the availability and physicians' compliance with clinical guidelines for the management of asthma is mandatory. In this survey, we found that the practice of physicians was suboptimal despite the recommendations in national and international asthma guidelines. These findings may be influenced by the low number of physicians and/or a lack of training. Furthermore, the findings revealed that although the physicians had good knowledge about asthma management in general, it did not reflect in their practice. Almost all the questions that reflect their practice were answered correctly by <70% of the participants. These are alarming results that mandated policymakers to implement a strategy to enhance the practice of physicians in the management of asthma.

In Jordan, the management of non-communicable diseases at the Ministry of Health is carried out through the primary healthcare (PHC) facilities, with spending on PHC services including asthma representing 16% of the allocated budget, and hence, it is a high burden. The major challenge facing the quality of health services is the lack of guiding clinical signs and national therapeutic guidelines. Furthermore, the PHC centers are crowded, and the physicians' workload is high (8, 9, 24). This could be a contributing factor to poor practice among physicians in Jordan.

Similar studies have demonstrated findings that are in accordance with our findings. A study by (25) revealed that only 28.6% of the investigated physicians (general practitioners) have adequate knowledge regarding asthma, and only 10.4% of them had adequate practice in asthma management (25). A cross-sectional study by (26) reported that asthma control in Jordan was achieved in 45.2% of the study sample (26). Several other studies also reported poor management of asthma. A study by (27) reported that only 30% of adult patients have achieved asthma control (27). Where poor asthma control in adolescence in Jordan was attributed to smoking (10), only 66% of physicians provide smoking cessation counseling and/or recommend cessation measures.

Furthermore, a large study in 2018 in the Middle East and North Africa reported that asthma was controlled in <30% of patients and it was attributed to poor adherence to prescribed medication, smoking, lack of medical insurance, and education level (28). Such results indicate that access to proper treatment, medication adherence, follow-ups by physicians, and education among healthcare providers and patients are mandatory to achieve better control of asthma (26).

The current study showed that practice significantly differed according to practitioner age, years of experience, and the number of asthma patients seen in their clinic. Logistic regression analysis indicated that physicians within the age group of 35–39 years [OR: 0.47 (CI: 0.24–0.90), *p* < 0.05] had a significant negative association with good practice; whereas senior physicians (aged above 50 years) had a significant and positive association with good practice [OR: 1.96 (CI: 1.03–3.75), *p* < 0.05]. Furthermore, those with more than 20 years of experience showed a positive pattern in practice [OD: 1.96 (CI: 1.02–3.74), *p* < 0.05]. Our findings are in accordance with Adeniyi et al. (17) who revealed a statistically significant difference in practice according to specialty (17). Hence, more experienced physicians who have a moderate number of patients could provide better practice. It is possible that the category of physicians who saw a high number of patients are under pressure, and that jeopardizes the level of practice provided to their patients due to their heavy workload. Several studies reported on barriers to providing optimal care to patients and compliance with guidelines that included lack of time and experience (29, 30).

The findings of this study identify the need to transform knowledge into practice. This could be achieved through professional education of physicians, constant reminders to physicians in a simple form, and clinical audit of practice (31–33). Approaches that involve tutorials, longer duration courses, and online peer discussion lead to improved knowledge transfer (34). However, education alone may not result in a change in physicians' practice (35). The education delivered should be enabling and reinforcing, aligning with the physicians' workflow. An effective means of knowledge transfer should be developed and implemented to improve the translation of guideline recommendations into care. However, such approaches may not be effective in the broader context in which clinical decisions are made, especially those pertinent to workload. Whilst physicians' competency remains a critical requirement for good practice, it may not be enough to presume that poor practice can simply be rectified through education (36). One study reported that there is an empirical association between physicians' workload, stress and poor practice (37). Therefore, there is a need for novel knowledge transfer approaches to induce behavioral and practice change.

### Strengths and Limitations

To the best of our knowledge, this is the first study in Jordan that investigated physicians' knowledge and practices regarding asthma management. One strength of the study is that we used previously validated assessment tools which enhanced the reliability and enabled comparison with other studies. Another strength is that using the online tool enabled us to contact physicians throughout Jordan throughout the time of the COVID-19 pandemic.

However, there are some limitations. The study design itself, a cross-sectional survey design, limited our ability to identify causality between study variables. In this study, we employed a quantitative methodology with pre-set responses, which might not have allowed physicians' views to provide varied but useful qualitative information. Due to the nature of the questionnaire distribution (which is an online questionnaire), we might have missed some of the target population who are not active on social media and other platforms. However, due to the current situation in the country which restricted the ability to distribute the questionnaire in person to physicians, an online survey was preferred to avoid the possibility of getting infected by COVID-19. Finally, we were not able to estimate the response rate for our questionnaire study, which might lead to a non-response bias, as we could not demonstrate how well the sample was drawn.

## Conclusions

Although physicians in Jordan demonstrated good knowledge pertinent to asthma management, there are practice gaps when compared with standard guidelines. Such a good level of knowledge does not appear to directly translate into good practice, which is reflected by the low level of asthma control among patients. The findings revealed an alarming trend in practice in Jordan as well as other countries using the same validated tool. There is an urgent need for novel knowledge transfer strategies that reinforce behavioral changes toward better practice; and which employ educational and professional development approaches, taking into account physicians' workloads.

## Data Availability Statement

The original contributions presented in the study are included in the article/supplementary material, further inquiries can be directed to the corresponding author/s.

## Author Contributions

The author confirms being the sole contributor of this work and has approved it for publication.

## Conflict of Interest

The author declares that the research was conducted in the absence of any commercial or financial relationships that could be construed as a potential conflict of interest.

## Publisher's Note

All claims expressed in this article are solely those of the authors and do not necessarily represent those of their affiliated organizations, or those of the publisher, the editors and the reviewers. Any product that may be evaluated in this article, or claim that may be made by its manufacturer, is not guaranteed or endorsed by the publisher.
